# Supplementary figures and images for: Control signal dimensionality depends on limb dynamics
Source: PLoS One. 2025 Apr 30;20(4):e0322092. doi: 10.1371/journal.pone.0322092 (PMC12043163; doi:10.1371/journal.pone.0322092)

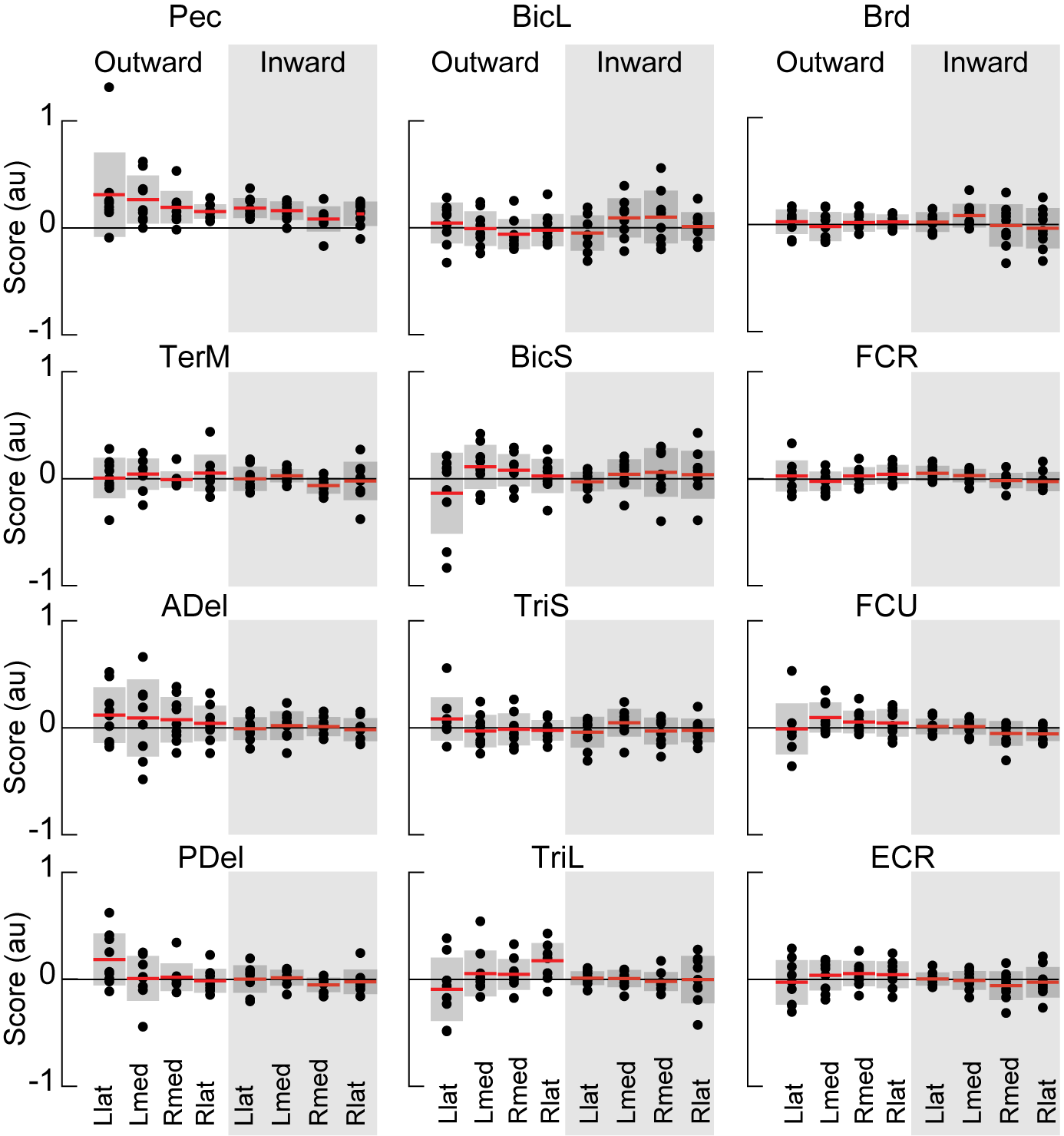

Supplement: S1 Fig — Dots show individual scores averaged across movement directions, red lines show mean values and grey boxes show standard deviations. EMG V1 and EMG V2 are the 1st and 2nd eigenvectors respectively obtained from EMG. Llat and Lmed indicate reaching with left arm in lateral and medial workspaces respectively; Rlat and Rmed indicate reaching with right arm in lateral and medial workspaces respectively. Muscle abbreviations: the clavicular head of pectoralis (Pec), teres major (TerM), anterior deltoid (ADel), posterior deltoid (PDel), the long and lateral heads of triceps (TriL and TriS), the short and long heads of biceps (BiS and BiL), brachioradialis (Brd), flexor carpi radialis (FCR), flexor carpi ulnaris (FCU), and extensor carpi radialis (ECR). (TIF) [file pone.0322092.s001.tif]

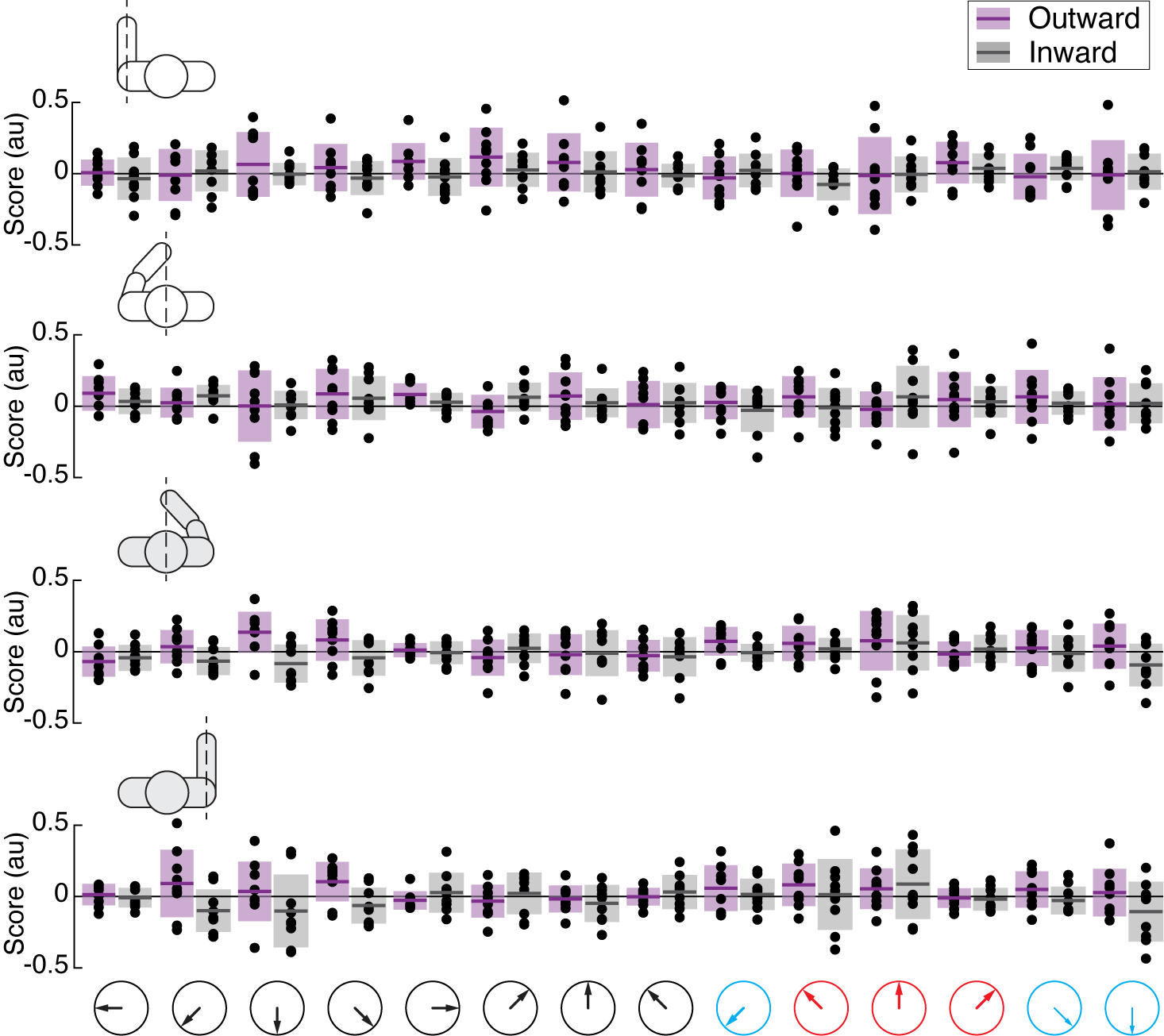

Supplement: S2 Fig — Data from the four conditions indicated by the pictograms are shown in plots arranged in rows. Dots show individual scores averaged across muscles, lines show mean values, shaded boxes show standard deviations, blue indicates outward reaches, and grey indicates inward reaches. Circles with arrows show reaching directions; black circles indicate reaching in the horizontal plane; blue circles indicate reaching downwards with gravity in the vertical plane; red circles indicate reaching upwards against gravity in the vertical plane. (TIF) [file pone.0322092.s002.tif]

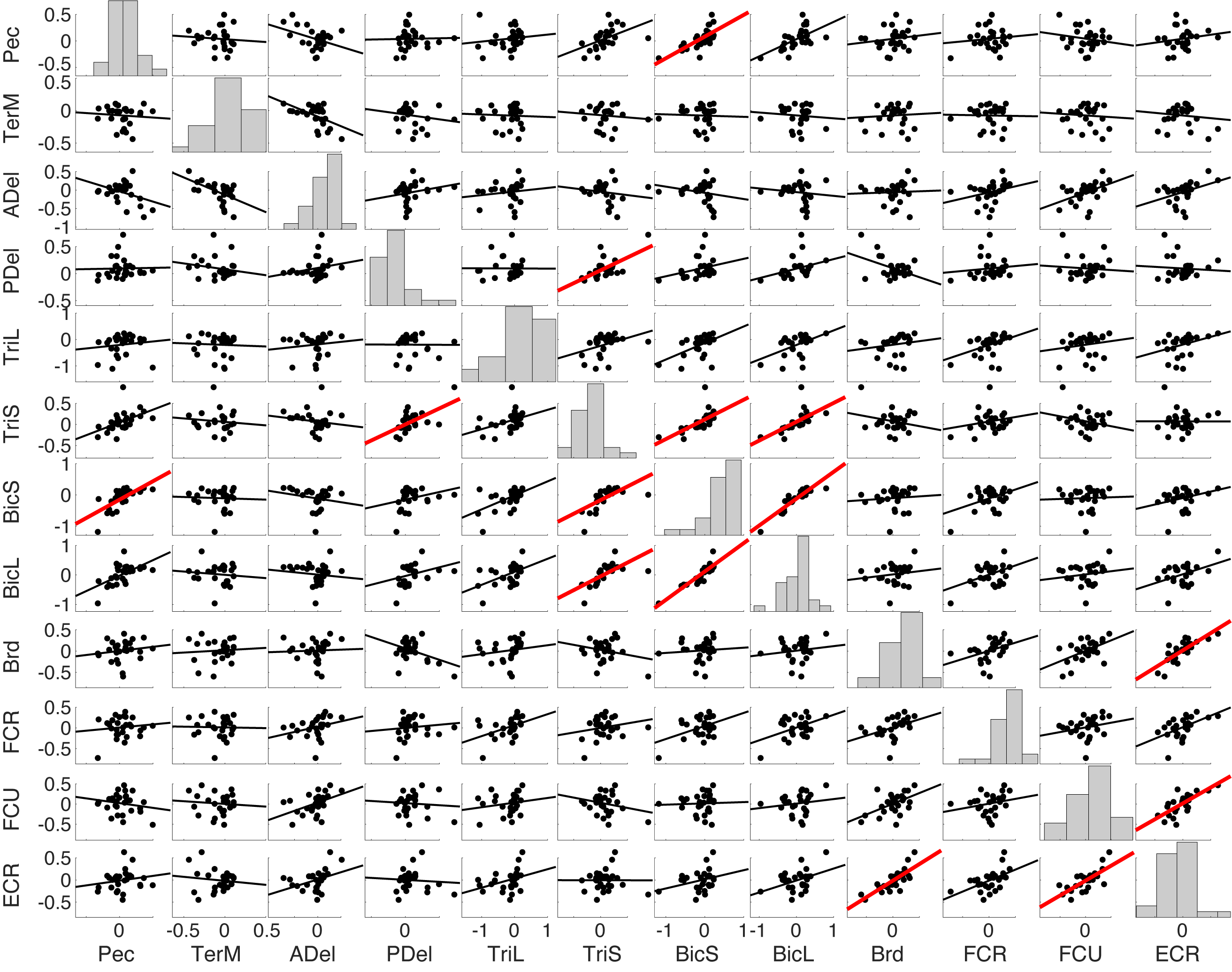

Supplement: S3 Fig — The coordinates of each dot represent principal component scores for two muscles for a single reaching direction, as in Fig 4B. Muscle abbreviations: the clavicular head of pectoralis (Pec), teres major (TerM), anterior deltoid (ADel), posterior deltoid (PDel), the long and lateral heads of triceps (TriL and TriS), the short and long heads of biceps (BiS and BiL), brachioradialis (Brd), flexor carpi radialis (FCR), flexor carpi ulnaris (FCU), and extensor carpi radialis (ECR). Histograms along the diagonal show the distribution of the scores for a given muscle across reaching directions. Solid lines show least-squares linear regression, and red lines indicate significant relationships with correction for family-wise error, the adjusted alpha = 0.0008. (TIF) [file pone.0322092.s003.tif]

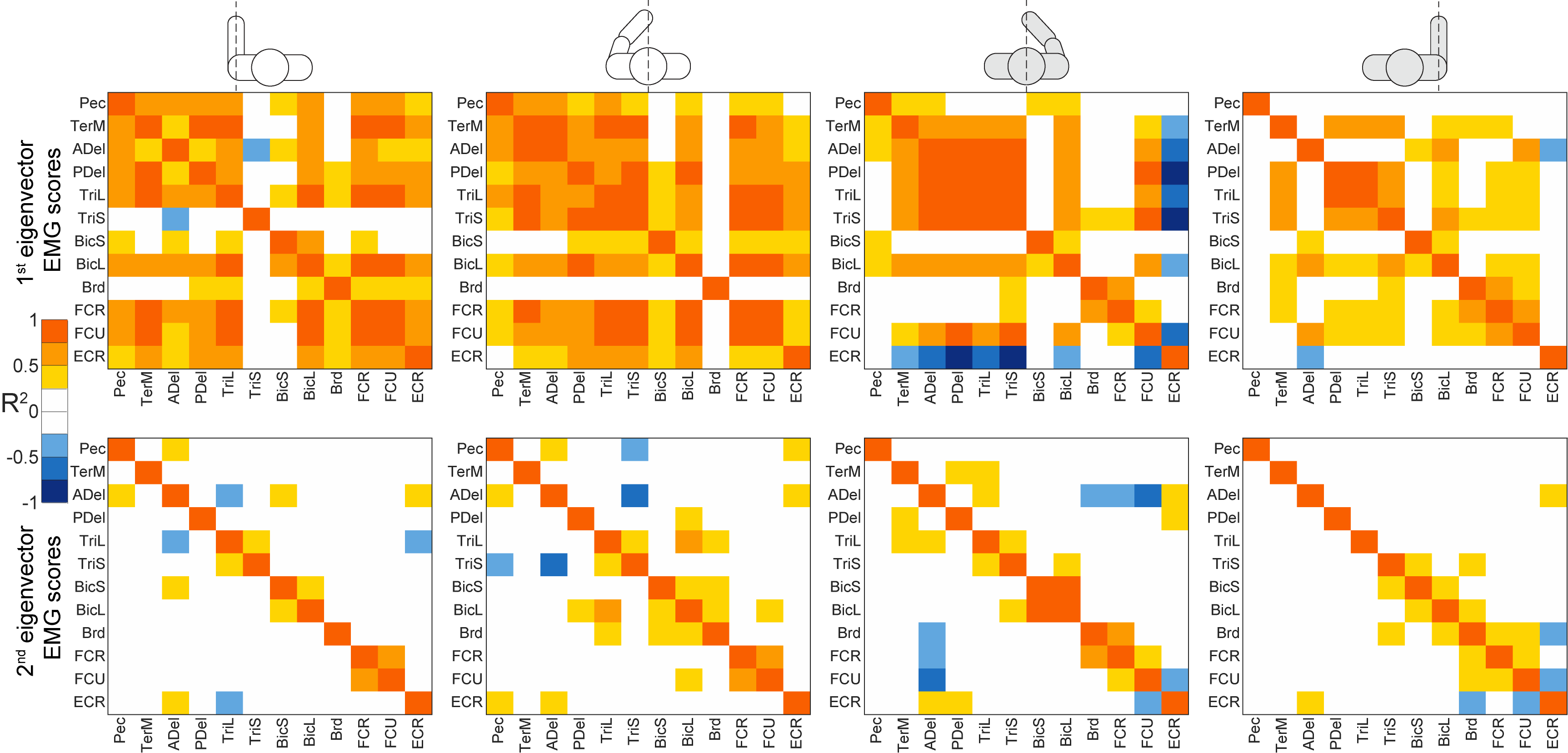

Supplement: S1 File — Heatmaps show coefficients of determination (R2). Red, orange, and darker blue colors represent moderate and strong relationships between scores for the EMG V1 (top row) and EMG V2 (bottom row) across reaching directions. Pictograms indicate conditions for left reaching in the lateral or medial workspace and for right reaching in the lateral or medial workspace. Muscles are abbreviated as follows: the clavicular head of pectoralis (Pec), teres major (TerM), anterior deltoid (ADel), posterior deltoid (PDel), the long and lateral heads of triceps (TriL and TriS), the short and long heads of biceps (BiS and BiL), brachioradialis (Brd), flexor carpi radialis (FCR), flexor carpi ulnaris (FCU), and extensor carpi radialis (ECR). (ZIP) [file pone.0322092.s004.zip › S1 File/S10_Fig.tif]

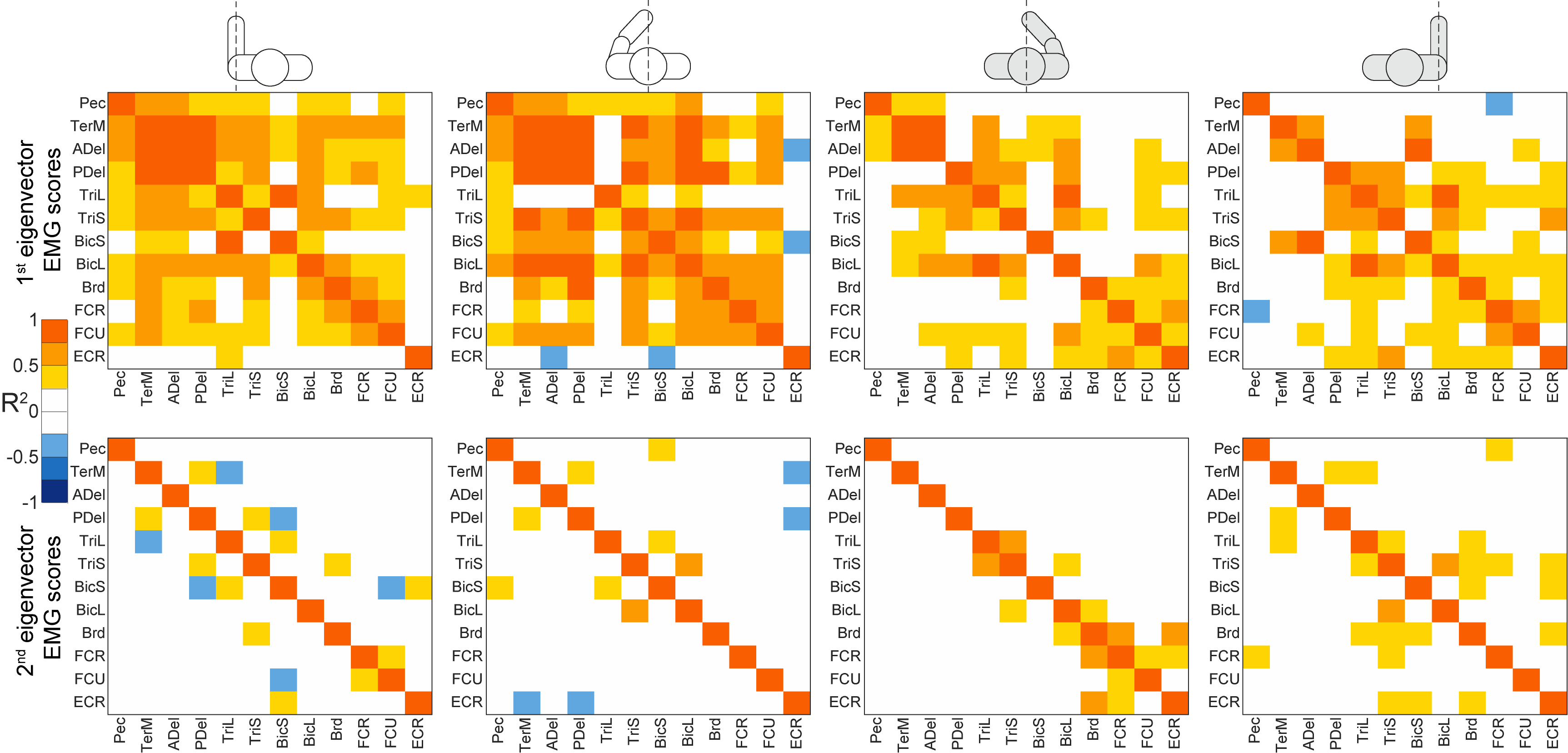

Supplement: S1 File — Heatmaps show coefficients of determination (R2). Red, orange, and darker blue colors represent moderate and strong relationships between scores for the EMG V1 (top row) and EMG V2 (bottom row) across reaching directions. Pictograms indicate conditions for left reaching in the lateral or medial workspace and for right reaching in the lateral or medial workspace. Muscles are abbreviated as follows: the clavicular head of pectoralis (Pec), teres major (TerM), anterior deltoid (ADel), posterior deltoid (PDel), the long and lateral heads of triceps (TriL and TriS), the short and long heads of biceps (BiS and BiL), brachioradialis (Brd), flexor carpi radialis (FCR), flexor carpi ulnaris (FCU), and extensor carpi radialis (ECR). (ZIP) [file pone.0322092.s004.zip › S1 File/S11_Fig.tif]

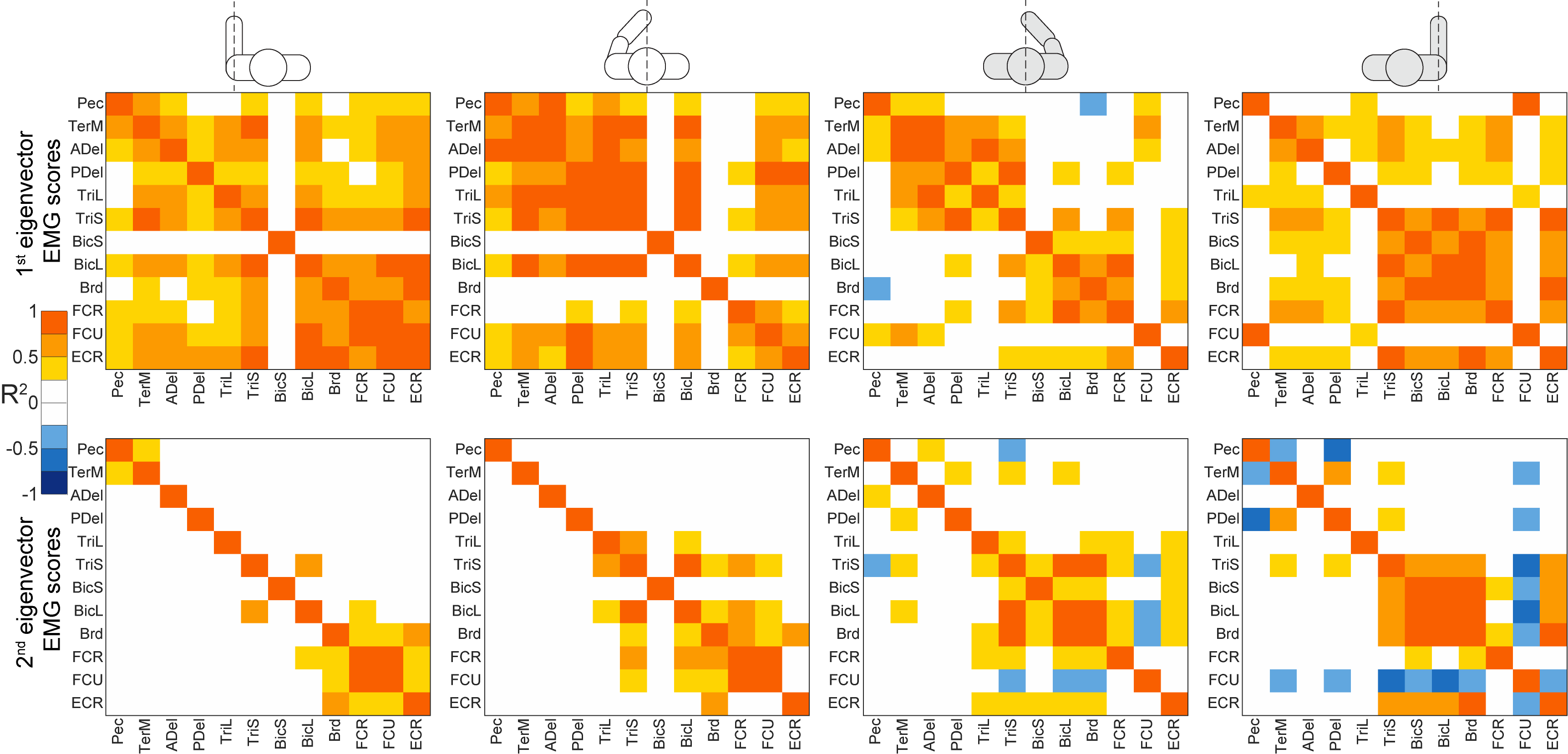

Supplement: S1 File — Heatmaps show coefficients of determination (R2). Red, orange, and darker blue colors represent moderate and strong relationships between scores for the EMG V1 (top row) and EMG V2 (bottom row) across reaching directions. Pictograms indicate conditions for left reaching in the lateral or medial workspace and for right reaching in the lateral or medial workspace. Muscles are abbreviated as follows: the clavicular head of pectoralis (Pec), teres major (TerM), anterior deltoid (ADel), posterior deltoid (PDel), the long and lateral heads of triceps (TriL and TriS), the short and long heads of biceps (BiS and BiL), brachioradialis (Brd), flexor carpi radialis (FCR), flexor carpi ulnaris (FCU), and extensor carpi radialis (ECR). (ZIP) [file pone.0322092.s004.zip › S1 File/S12_Fig.tif]

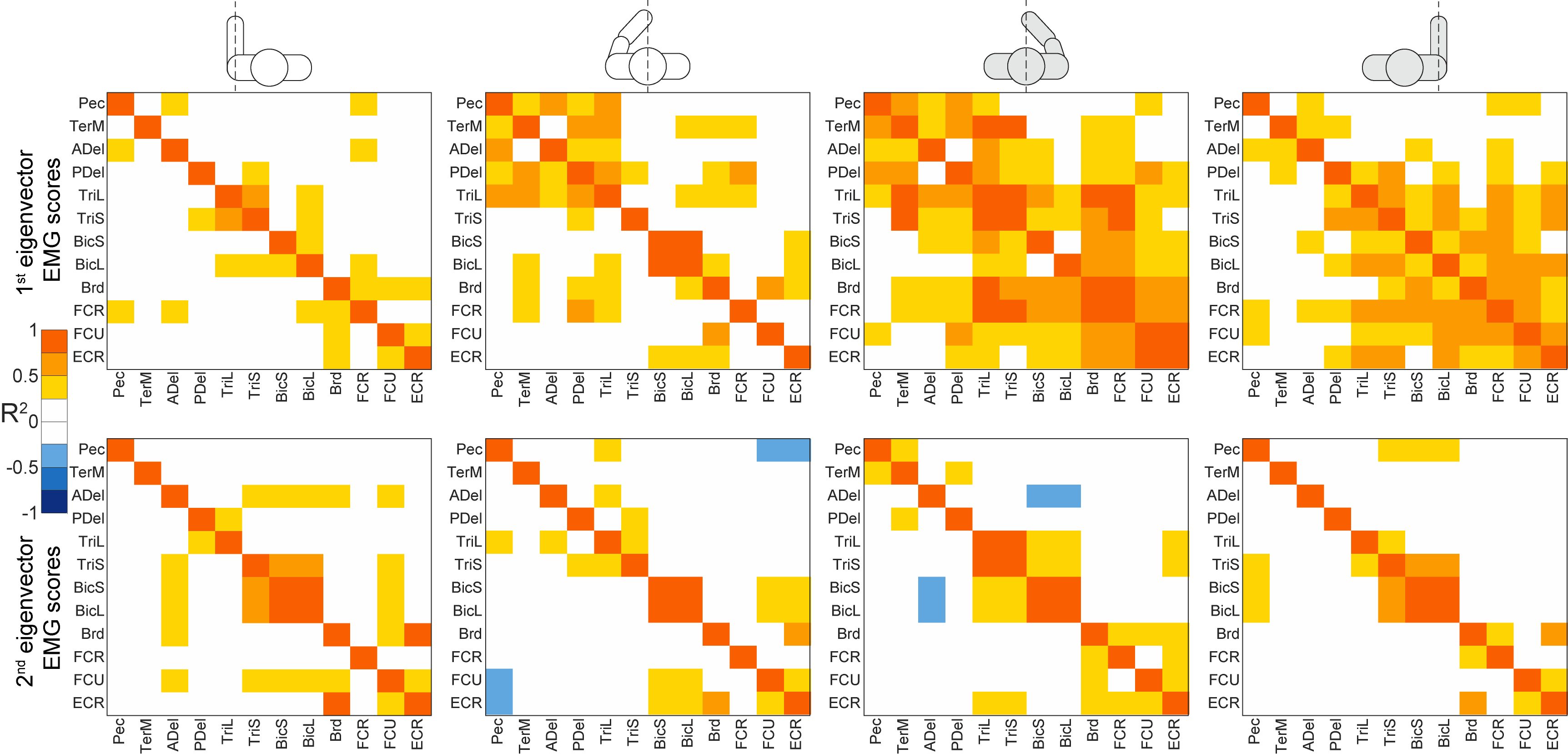

Supplement: S1 File — Heatmaps show coefficients of determination (R2). Red, orange, and darker blue colors represent moderate and strong relationships between scores for the EMG V1 (top row) and EMG V2 (bottom row) across reaching directions. Pictograms indicate conditions for left reaching in the lateral or medial workspace and for right reaching in the lateral or medial workspace. Muscles are abbreviated as follows: the clavicular head of pectoralis (Pec), teres major (TerM), anterior deltoid (ADel), posterior deltoid (PDel), the long and lateral heads of triceps (TriL and TriS), the short and long heads of biceps (BiS and BiL), brachioradialis (Brd), flexor carpi radialis (FCR), flexor carpi ulnaris (FCU), and extensor carpi radialis (ECR). (ZIP) [file pone.0322092.s004.zip › S1 File/S4_Fig.tif]

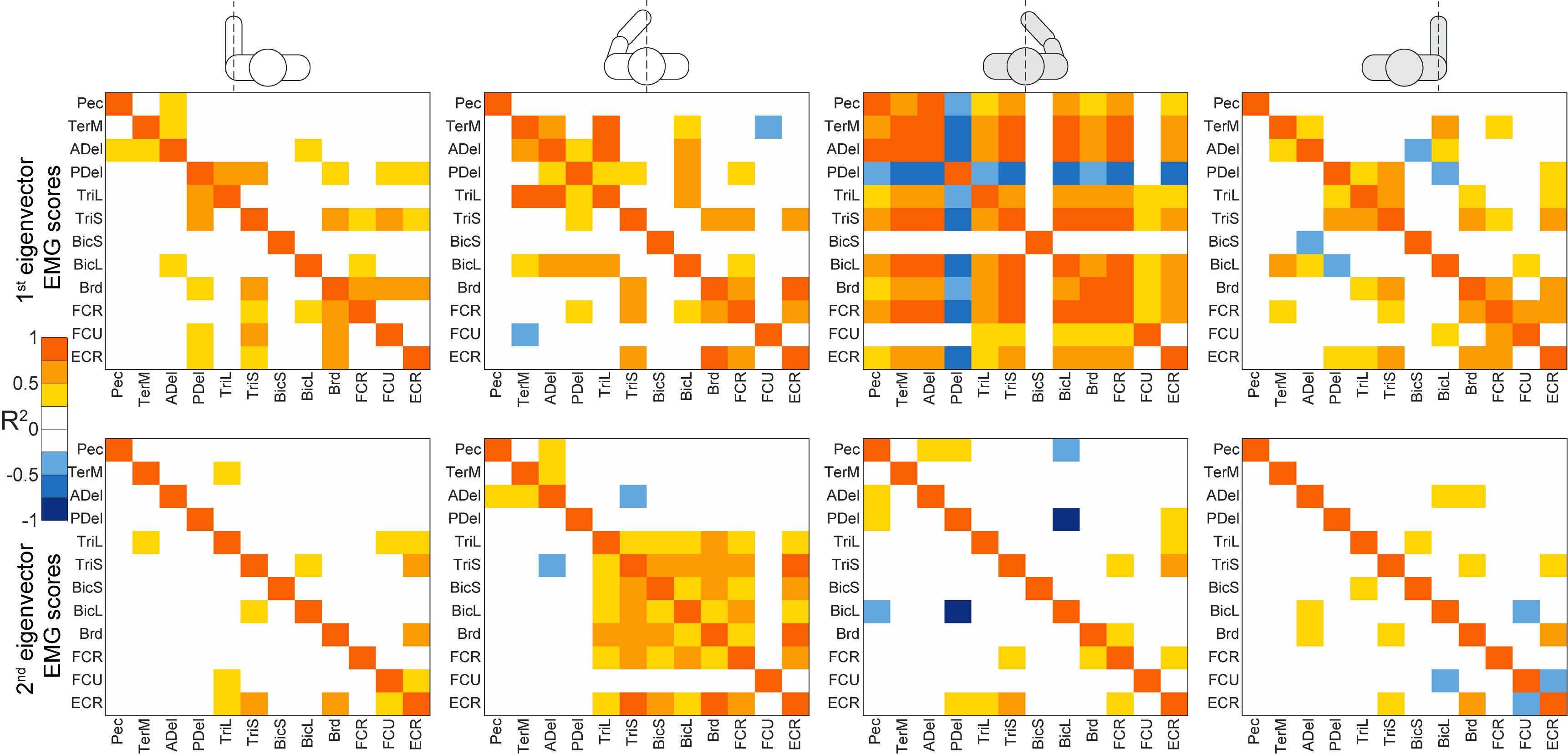

Supplement: S1 File — Heatmaps show coefficients of determination (R2). Red, orange, and darker blue colors represent moderate and strong relationships between scores for the EMG V1 (top row) and EMG V2 (bottom row) across reaching directions. Pictograms indicate conditions for left reaching in the lateral or medial workspace and for right reaching in the lateral or medial workspace. Muscles are abbreviated as follows: the clavicular head of pectoralis (Pec), teres major (TerM), anterior deltoid (ADel), posterior deltoid (PDel), the long and lateral heads of triceps (TriL and TriS), the short and long heads of biceps (BiS and BiL), brachioradialis (Brd), flexor carpi radialis (FCR), flexor carpi ulnaris (FCU), and extensor carpi radialis (ECR). (ZIP) [file pone.0322092.s004.zip › S1 File/S5_Fig.tif]

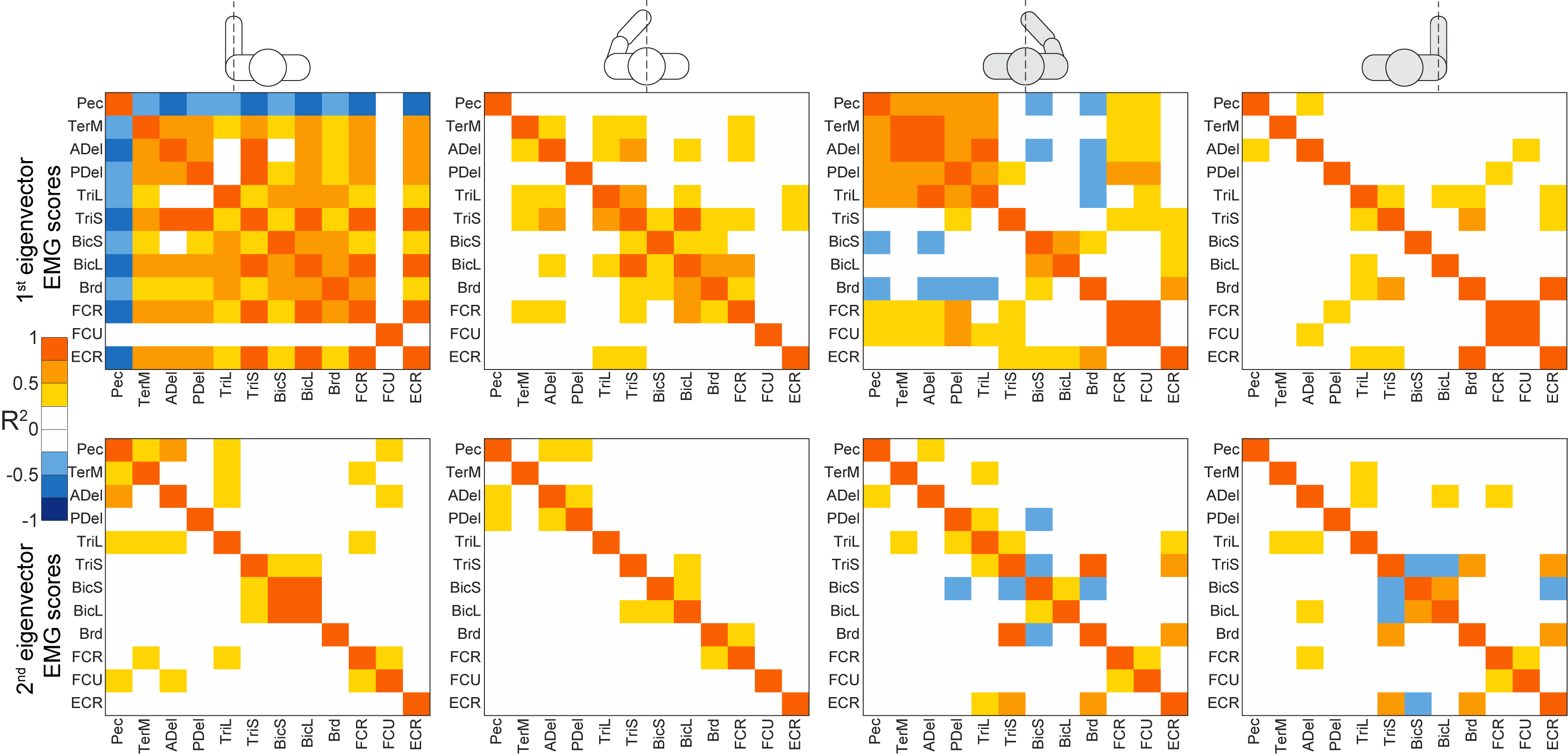

Supplement: S1 File — Heatmaps show coefficients of determination (R2). Red, orange, and darker blue colors represent moderate and strong relationships between scores for the EMG V1 (top row) and EMG V2 (bottom row) across reaching directions. Pictograms indicate conditions for left reaching in the lateral or medial workspace and for right reaching in the lateral or medial workspace. Muscles are abbreviated as follows: the clavicular head of pectoralis (Pec), teres major (TerM), anterior deltoid (ADel), posterior deltoid (PDel), the long and lateral heads of triceps (TriL and TriS), the short and long heads of biceps (BiS and BiL), brachioradialis (Brd), flexor carpi radialis (FCR), flexor carpi ulnaris (FCU), and extensor carpi radialis (ECR). (ZIP) [file pone.0322092.s004.zip › S1 File/S6_Fig.tif]

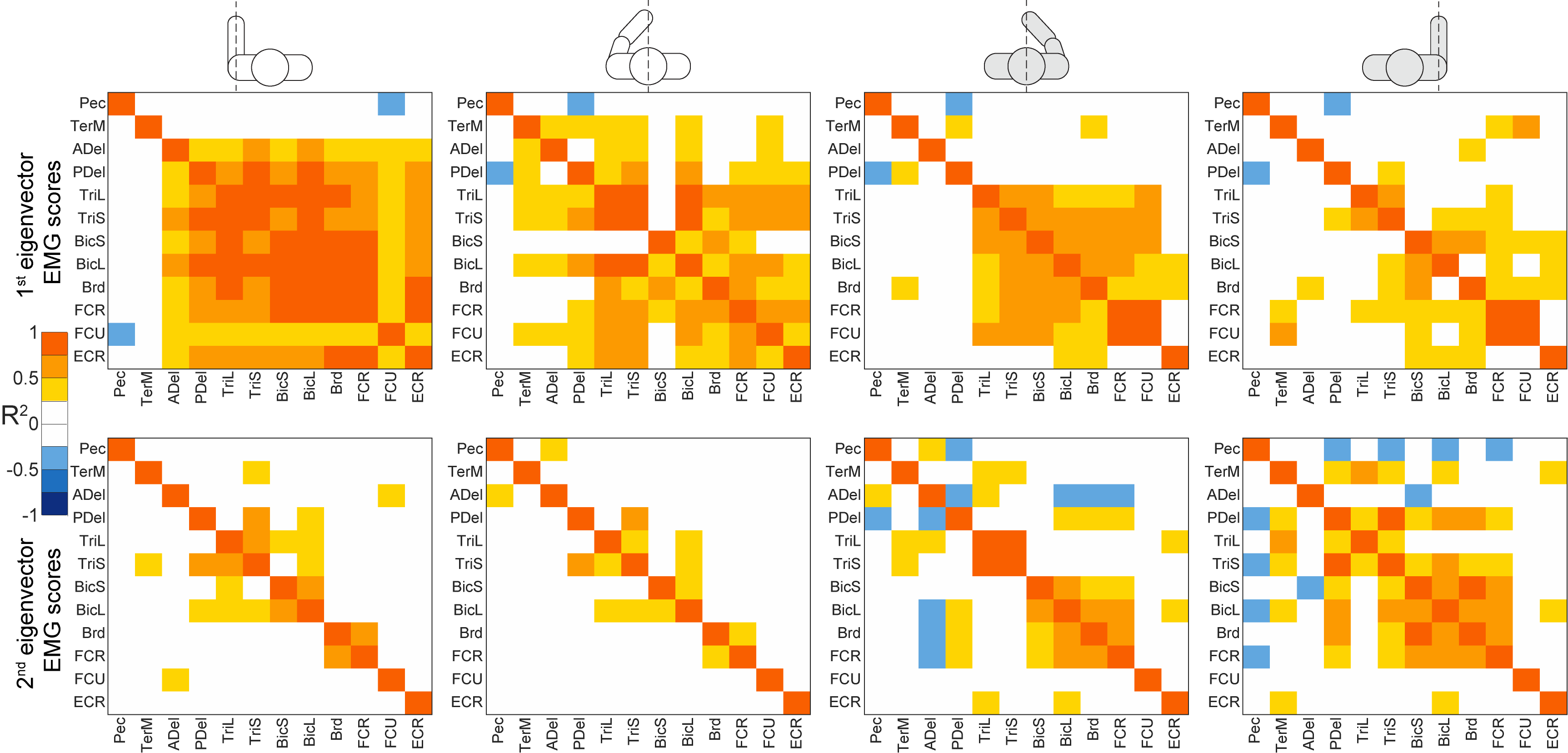

Supplement: S1 File — Heatmaps show coefficients of determination (R2). Red, orange, and darker blue colors represent moderate and strong relationships between scores for the EMG V1 (top row) and EMG V2 (bottom row) across reaching directions. Pictograms indicate conditions for left reaching in the lateral or medial workspace and for right reaching in the lateral or medial workspace. Muscles are abbreviated as follows: the clavicular head of pectoralis (Pec), teres major (TerM), anterior deltoid (ADel), posterior deltoid (PDel), the long and lateral heads of triceps (TriL and TriS), the short and long heads of biceps (BiS and BiL), brachioradialis (Brd), flexor carpi radialis (FCR), flexor carpi ulnaris (FCU), and extensor carpi radialis (ECR). (ZIP) [file pone.0322092.s004.zip › S1 File/S7_Fig.tif]

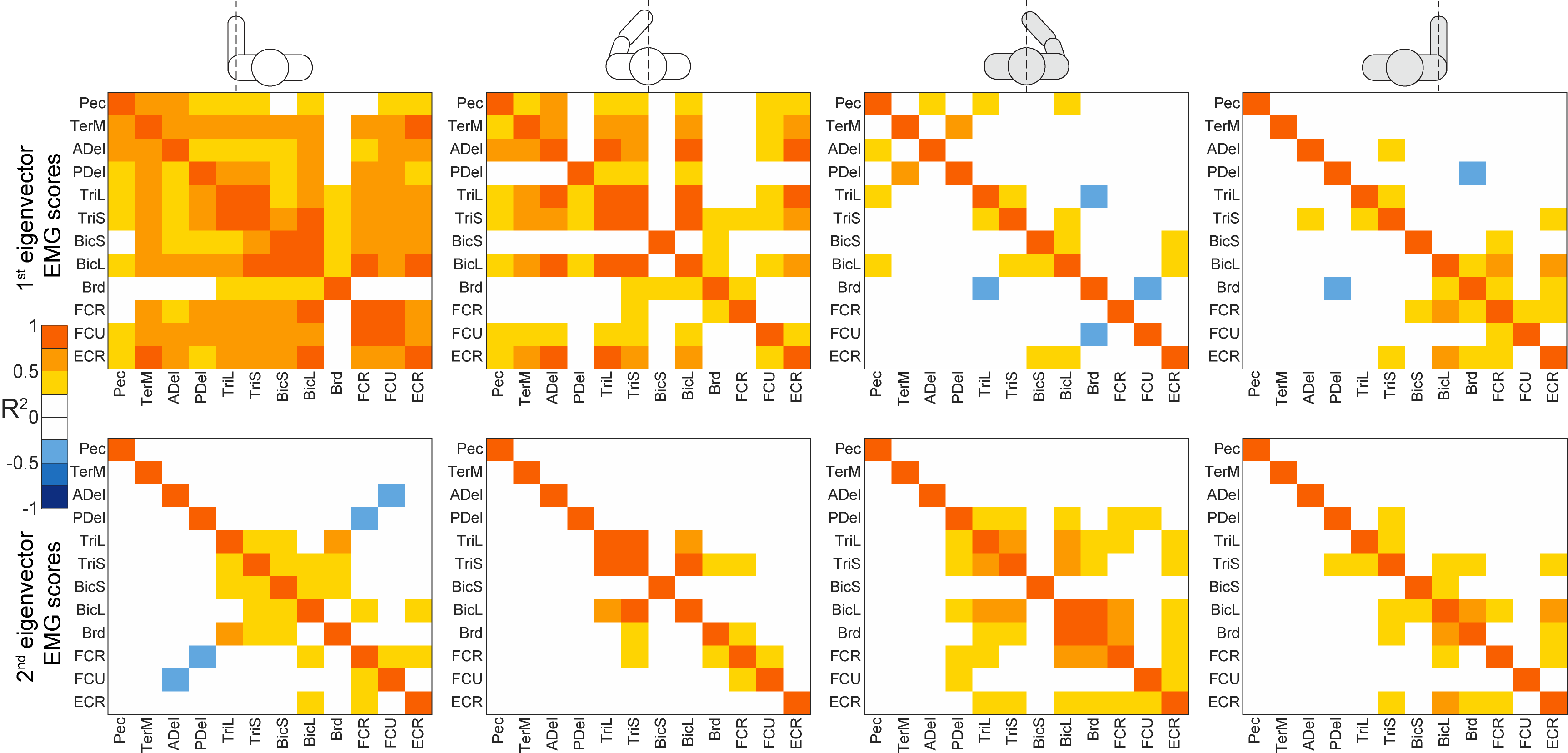

Supplement: S1 File — Heatmaps show coefficients of determination (R2). Red, orange, and darker blue colors represent moderate and strong relationships between scores for the EMG V1 (top row) and EMG V2 (bottom row) across reaching directions. Pictograms indicate conditions for left reaching in the lateral or medial workspace and for right reaching in the lateral or medial workspace. Muscles are abbreviated as follows: the clavicular head of pectoralis (Pec), teres major (TerM), anterior deltoid (ADel), posterior deltoid (PDel), the long and lateral heads of triceps (TriL and TriS), the short and long heads of biceps (BiS and BiL), brachioradialis (Brd), flexor carpi radialis (FCR), flexor carpi ulnaris (FCU), and extensor carpi radialis (ECR). (ZIP) [file pone.0322092.s004.zip › S1 File/S8_Fig.tif]

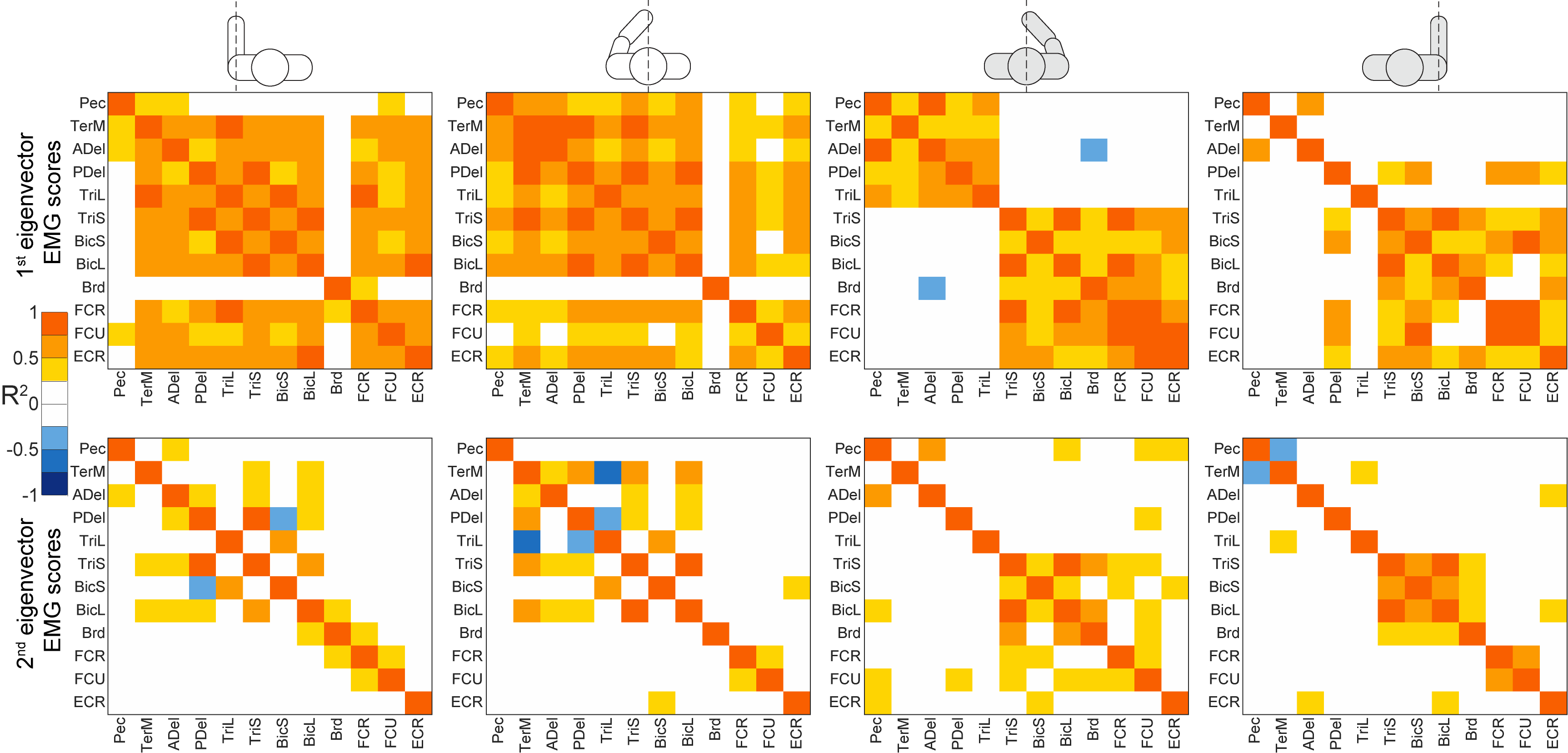

Supplement: S1 File — Heatmaps show coefficients of determination (R2). Red, orange, and darker blue colors represent moderate and strong relationships between scores for the EMG V1 (top row) and EMG V2 (bottom row) across reaching directions. Pictograms indicate conditions for left reaching in the lateral or medial workspace and for right reaching in the lateral or medial workspace. Muscles are abbreviated as follows: the clavicular head of pectoralis (Pec), teres major (TerM), anterior deltoid (ADel), posterior deltoid (PDel), the long and lateral heads of triceps (TriL and TriS), the short and long heads of biceps (BiS and BiL), brachioradialis (Brd), flexor carpi radialis (FCR), flexor carpi ulnaris (FCU), and extensor carpi radialis (ECR). (ZIP) [file pone.0322092.s004.zip › S1 File/S9_Fig.tif]
