# Supplementary material for: Control signal dimensionality depends on limb dynamics
Source: PLoS One. 2025 Apr 30;20(4):e0322092. doi: 10.1371/journal.pone.0322092 (PMC12043163; doi:10.1371/journal.pone.0322092)
Supplement: S1 Table — (PDF) [file pone.0322092.s005.pdf]

Table S1. Post-hoc differences in dynamic score between reaching directions.

Target		MSE ± SE	*p* value
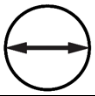	Horizontal plane, left – right	0.0131 ± 0.0108	0.9916
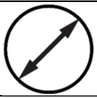	Horizontal plane, toward – away from body	0.006 ± 0.0178	1.0000
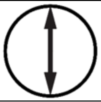	Horizontal plane, back – forward	-0.0135 ± 0.0233	1.0000
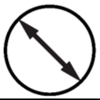	Horizontal plane, lateral – medial	0.016 ± 0.0129	0.9907
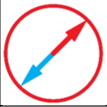	Vertical plane, down toward the body – up away from the body	-0.0087 ± 0.0143	1.0000
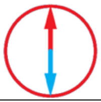	Vertical plane, down – up	-0.0018 ± 0.0191	1.0000
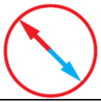	Vertical plane, up toward the body – down away from the body	-0.0044 ± 0.0255	1.0000

MSE – mean squared error, SE – standard error of the mean. Bold *p* values show significant differences with family-wise correction. Target location pictograms and colors are as described in Methods and shown in Fig 8.
