# Supplementary material for: Control signal dimensionality depends on limb dynamics
Source: PLoS One. 2025 Apr 30;20(4):e0322092. doi: 10.1371/journal.pone.0322092 (PMC12043163; doi:10.1371/journal.pone.0322092)
Supplement: S2 Table — (PDF) [file pone.0322092.s006.pdf]

Table S2. Post-hoc differences in dynamic score between muscles.

Muscle	MSE ± SE	*p* value	Muscle	MSE ± SE	*p* value
**Pec vs TerM**	-0.0305 ± 0.0230	0.968	**BicL vs BicS**	0.0125 ± 0.0257	1.000
**Pec vs ADel**	0.0007 ± 0.0184	1.000	**BicL vs TriL**	0.0274 ± 0.0218	0.978
**Pec vs PDel**	-0.0542 ± 0.0182	0.167	**BicL vs TriS**	0.0160 ± 0.0139	0.989
**Pec vs BicL**	-0.0548 ± 0.0218	0.372	**BicL vs Brd**	0.0127 ± 0.0181	1.000
**Pec vs BicS**	-0.0424 ± 0.0278	0.921	**BicL vs ECR**	0.0447 ± 0.0140	0.112
**Pec vs TriL**	-0.0274 ± 0.0229	0.985	**BicL vs FCR**	0.0569 ± 0.0188	0.154
**Pec vs TriS**	-0.0388 ± 0.0212	0.789	**BicL vs FCU**	0.0340 ± 0.0210	0.888
**Pec vs Brd**	-0.0422 ± 0.0253	0.868			
**Pec vs ECR**	-0.0102 ± 0.0214	1.000	**BicS vs TriL**	0.0150 ± 0.0193	1.000
**Pec vs FCR**	0.0021 ± 0.0198	1.000	**BicS vs TriS**	0.0035 ± 0.0310	1.000
**Pec vs FCU**	-0.0209 ± 0.0175	0.985	**BicS vs Brd**	0.0002 ± 0.0253	1.000
			**BicS vs ECR**	0.0322 ± 0.0258	0.979
**TerM vs ADel**	0.0312 ± 0.0237	0.969	**BicS vs FCR**	0.0444 ± 0.0285	0.909
**TerM vs PDel**	-0.0238 ± 0.0174	0.961	**BicS vs FCU**	0.0215 ± 0.0291	1.000
**TerM vs BicL**	-0.0244 ± 0.0194	0.978			
**TerM vs BicS**	-0.0119 ± 0.0294	1.000	**TriL vs TriS**	-0.0114 ± 0.0249	1.000
**TerM vs TriL**	0.0030 ± 0.0203	1.000	**TriL vs Brd**	-0.0148 ± 0.0212	1.000
**TerM vs TriS**	-0.0084 ± 0.0191	1.000	**TriL vs ECR**	0.0172 ± 0.0203	0.999
**TerM vs Brd**	-0.0117 ± 0.0231	1.000	**TriL vs FCR**	0.0295 ± 0.0216	0.961
**TerM vs ECR**	0.0203 ± 0.0207	0.997	**TriL vs FCU**	0.0065 ± 0.0227	1.000
**TerM vs FCR**	0.0325 ± 0.0178	0.793			
**TerM vs FCU**	0.0096 ± 0.0173	1.000			
**ADel vs PDel**	-0.0550 ± 0.0224	0.407	**TriS vs Brd**	-0.0034 ± 0.023	1.000
**ADel vs BicL**	-0.0556 ± 0.0227	0.410	**TriS vs ECR**	0.0286 ± 0.0173	0.875
**ADel vs BicS**	-0.0431 ± 0.0260	0.872	**TriS vs FCR**	0.0409 ± 0.0193	0.613
**ADel vs TriL**	-0.0281 ± 0.0209	0.964	**TriS vs FCU**	0.0180 ± 0.0206	0.999
**ADel vs TriS**	-0.0396 ± 0.0243	0.885			
**ADel vs Brd**	-0.0429 ± 0.0239	0.808	**Brd vs ECR**	0.0320 ± 0.0123	0.331
**ADel vs ECR**	-0.0109 ± 0.0206	1.000	**Brd vs FCR**	0.0442 ± 0.0148	0.166
**ADel vs FCR**	0.0013 ± 0.0201	1.000	**Brd vs FCU**	0.0213 ± 0.0222	0.997
**ADel vs FCU**	-0.0216 ± 0.0221	0.997			
**PDel vs BicL**	-0.0006 ± 0.0193	1.000	**ECR vs FCR**	0.0122 ± 0.0148	0.999
**PDel vs BicS**	0.0119 ± 0.0331	1.000	**ECR vs FCU**	-0.0107 ± 0.0200	1.000
**PDel vs TriL**	0.0268 ± 0.0251	0.994	**FCR vs FCU**	-0.0229 ± 0.0152	0.924
**PDel vs TriS**	0.0154 ± 0.0145	0.994			
**PDel vs Brd**	0.0121 ± 0.0253	1.000			
**PDel vs ECR**	0.0440 ± 0.0217	0.674			
**PDel vs FCR**	0.0563 ± 0.0168	0.079			
**PDel vs FCU**	0.0334 ± 0.0172	0.726			

MSE – mean squared error, SE – standard error of the mean. Bold *p* values show significant differences with family-wise correction. Muscle abbreviations: the clavicular head of pectoralis (Pec), teres major (TerM), anterior deltoid (ADel), posterior deltoid (PDel), the long and lateral heads of triceps (TriL and TriS), the short and long heads of biceps (BiS and BiL), brachioradialis (Brd), flexor carpi radialis (FCR), flexor carpi ulnaris (FCU), and extensor carpi radialis (ECR).
