# Supplementary material for: Control signal dimensionality depends on limb dynamics
Source: PLoS One. 2025 Apr 30;20(4):e0322092. doi: 10.1371/journal.pone.0322092 (PMC12043163; doi:10.1371/journal.pone.0322092)
Supplement: S1 Text — (PDF) [file pone.0322092.s008.pdf]

West Virginia University
Office of Research Integrity and Compliance
886 CHESNUT RIDGE ROAD MORGANTOWN, WV 26506

Approval Letter Renewal

**Action Date**	03/21/2017
**To**	Valeriya Gritsenko
**From**	WVU Office of Research Integrity and Compliance
**Initial Approval Date**	03/12/2014
**Last Approval Date**	03/21/2017
**Expiration Date**	03/20/2018
**Subject**	Continuing Review of Approved Protocol
**Protocol Number**	1311129283R003
**Title**	A New Quantitative Biomechanical Method for Motor Assessment of Disability

---

The continuing review/renewal referenced above has been reviewed and approved by the Expedited Board of the West Virginia University Institutional Review Board (IRB).

- Category 4. Collection of data through noninvasive procedures (not involving general anesthesia or sedation) routinely employed in clinical practice, excluding procedures involving x-rays or microwaves. Where medical devices are employed, they must be cleared/approved for marketing. (Studies intended to evaluate the safety and effectiveness of the medical device are not generally eligible for expedited review, including studies of cleared medical devices for new indications.) Examples: (a) physical sensors that are applied either to the surface of the body or at a distance and do not involve input of significant amounts of energy into the subject or an invasion of the subjects privacy; (b) weighing or testing sensory acuity; (c) magnetic resonance imaging; (d) electrocardiography, electroencephalography, thermography, detection of naturally occurring radioactivity, electroretinography, ultrasound, diagnostic infrared imaging, doppler blood flow, and echocardiography; (e) moderate exercise, muscular strength testing, body composition assessment, and flexibility testing where appropriate given the age, weight, and health of the individual.
- Category 6. Collection of data from voice, video, digital, or image recordings made for research purposes.

Documents reviewed and/or approved as part of this submission:

Questionnaire.pdf: 2014-01-22-05:00

IRB approval letter.pdf: 2014-01-23-05:00

Recruitment flyer-With Picturesv2(1).pdf: 2015-04-09-04:00

FormAmendment Form .docx: 2015-04-09-04:00

Consent Form 031715.pdf: 2015-04-09-04:00

IRB Response 6.11.15.pdf: 2015-06-11-04:00

HIPAA Waiver Form KC1311129283.docx: 2015-06-24-04:00

Screening Checklist.docx: 2015-06-24-04:00

IRB Response 4.4.16.docx: 2016-04-04-04:00

Full protocol.pdf: 2014-01-22-05:00

Documents for use in this study are available in the WVUkc system in the Notes and Attachments section of your protocol.

The Office of Research Integrity and Compliance is here to provide assistance to you from the initial submission of an IRB protocol and all subsequent activity. Please feel free to contact us by phone at 304.293.7073 with any question you may have. Thank you.

WVU Office of Research Integrity and Compliance

Date:03/21/2017

Signed:

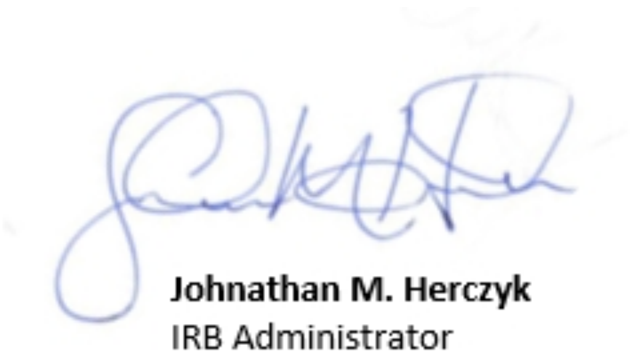

[illegible] wing regulations apply:

[illegible] encountered in this research study must be reported to the

2. Any modifications to the study protocol or informed consent form must be reviewed and approved by the IRB prior to implementation via submission of an amendment.

3. You may not use a modified informed consent form until it has been approved and validated by the IRB.

WestVirginiaUniversity
Office of Research Integrity and Compliance

Approval Letter Expedited

**Action Date**	06/22/2015
**To**	Valeriya Gritsenko
**From**	WVU Office of Research Integrity and Compliance
**Approval Date**	06/22/2015
**Expiration Date**	04/05/2016
**Subject**	Protocol Approval Letter
**Protocol Number**	1311129283A004
**Title**	A New Quantitative Biomechanical Method for Motor Assessment of Disability

---

The above-referenced research study was reviewed by the West Virginia University Institutional Review Board IRB and was approved in accordance with 46 CFR 46.101b.

It has been determined that this study is of minimal risk and meets the criteria as defined by the expedited categories listed below:

- Erican Blackwell will be added as a co-investigator and Kimberly Glover will be added as a member of the study personnel. A HIPAA waiver form and screening checklist will be added as attachments for patient recruitment purposes. The HIPAA questionnaire section will be updated to reflect the HIPAA waiver.

Documents reviewed and/or approved as part of this submission:

Full protocol.pdf: 2014-01-22-05:00

Questionnaire.pdf: 2014-01-22-05:00

IRB approval letter.pdf: 2014-01-23-05:00

Recruitment flyer-With Picturesv2(1).pdf: 2015-04-09-04:00

FormAmendment Form .docx: 2015-04-09-04:00

Consent Form 031715.pdf: 2015-04-09-04:00

HIPAA Waiver Form KC1311129283.docx: 2015-05-27-04:00

Screening Checklist.docx: 2015-05-27-04:00

IRB Response 6.11.15.pdf: 2015-06-11-04:00

Documents for use in this study are available in the WVUkc system in the Notes and Attachments section of your protocol.

The Office of Research Integrity and Compliance is here to provide assistance to you from the initial submission of an IRB protocol and all subsequent activity. Please feel free to contact us by phone at 304.293.7073 with any question you may have. Thank you.

WVU Office of Research Integrity and Compliance

Date:06/22/2015

Signed:

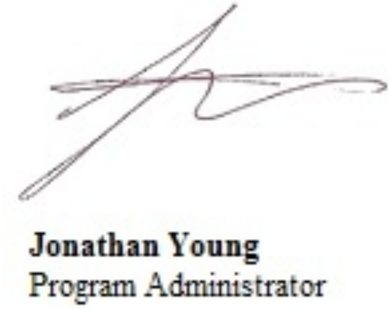

[illegible]ct research, the following regulations apply:

1. Unanticipated or serious adverse events/side effects encountered in this research study must be reported to the IRB within five (5) days via the Notify IRB action.

2. Any modifications to the study protocol or informed consent form must be reviewed and approved by the IRB prior to implementation via submission of an amendment.

3. You may not use a modified informed consent form until it has been approved and validated by the IRB.

WestVirginiaUniversity
Office of Research Integrity and Compliance

Approval Letter Expedited

**To**	Valeriya Gritsenko
**From**	WVU Office of Research Integrity and Compliance
**Action Date**	04/06/2015
**Approval Period**	04/06/2015 **Expiration Date** 04/05/2016
**Subject**	Protocol Approval Letter
**Protocol Number**	1311129283R001
**Title**	A New Quantitative Biomechanical Method for Motor Assessment of Disability

---

The above-referenced research study was reviewed by the West Virginia University Institutional Review Board IRB and was approved in accordance with 46 CFR 46.101b.

It has been determined that this study is of minimal risk and meets the criteria as defined by the expedited categories listed below:

- Category 4. Collection of data through noninvasive procedures (not involving general anesthesia or sedation) routinely employed in clinical practice, excluding procedures involving x-rays or microwaves. Where medical devices are employed, they must be cleared/approved for marketing. (Studies intended to evaluate the safety and effectiveness of the medical device are not generally eligible for expedited review, including studies of cleared medical devices for new indications.) Examples: (a) physical sensors that are applied either to the surface of the body or at a distance and do not involve input of significant amounts of energy into the subject or an invasion of the subjects privacy; (b) weighing or testing sensory acuity; (c) magnetic resonance imaging; (d) electrocardiography, electroencephalography, thermography, detection of naturally occurring radioactivity, electroretinography, ultrasound, diagnostic infrared imaging, doppler blood flow, and echocardiography; (e) moderate exercise, muscular strength testing, body composition assessment, and flexibility testing where appropriate given the age, weight, and health of the individual.
- Category 6. Collection of data from voice, video, digital, or image recordings made for research purposes.

Documents for use in this study are available in the WVUkc system in the Notes and Attachments section of your protocol.

The Office of Research Integrity and Compliance is here to provide assistance to you from the initial submission of an IRB protocol and all subsequent activity. Please feel free to contact us by phone at 304.293.7073 with any question you may have. Thank you.

WVU Office of Research Integrity and Compliance

Date: 04/06/2015

Signed:

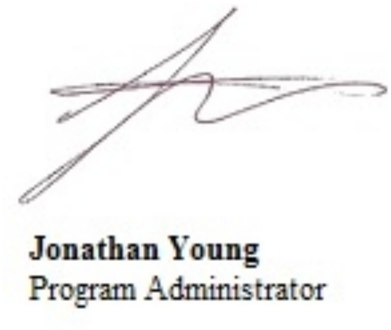

[illegible]ct research, the following regulations apply:

1. Unanticipated or serious adverse events/side effects encountered in this research study must be reported to the IRB within five (5) days via the Notify IRB action.

2. Any modifications to the study protocol or informed consent form must be reviewed and approved by the IRB prior to implementation via submission of an amendment.

3. You may not use a modified informed consent form until it has been approved and validated by the IRB.

West Virginia University
Office of Research Integrity and Compliance

Approval Letter Expedited

**To**	Valeriya Gritsenko
**From**	WVU Office of Research Integrity and Compliance
**Action Date**	12/05/2014
**Approval Period**	12/04/2014 **Expiration Date** 03/11/2015
**Subject**	Protocol Approval Letter
**Protocol Number**	1311129283A002
**Title**	A New Quantitative Biomechanical Method for Motor Assessment of Disability

---

The above-referenced research study was reviewed by the West Virginia University Institutional Review Board IRB and was approved in accordance with 46 CFR 46.101b.

It has been determined that this study is of minimal risk and meets the criteria as defined by the expedited categories listed below:

- Added 3 new study personnel: S. Collins, A. Adcock, and J. Brick The new sponsor is now listed on the consent form Added HIPPA Waver of Research Authorization that will be used by S. Collins and her team at WVCTRU to recruit subjects.

Documents for use in this study are available in the WVUkc system in the Notes and Attachments section of your protocol.

The Office of Research Integrity and Compliance is here to provide assistance to you from the initial submission of an IRB protocol and all subsequent activity. Please feel free to contact us by phone at 304.293.7073 with any question you may have. Thank you.

WVU Office of Research Integrity and Compliance

Date: 12/05/2014

Signed:

Lilo Ast

Lilo Ast
Senior Program Coordinator

Once you begin your human subject research, the following regulations apply:

1. Unanticipated or serious adverse events/side effects encountered in this research study must be reported to the IRB within five (5) days via the Notify IRB action.

2. Any modifications to the study protocol or informed consent form must be reviewed and approved by the IRB prior to implementation via submission of an amendment.

3. You may not use a modified informed consent form until it has been approved and validated by the IRB.
