# Supplementary material for: Control signal dimensionality depends on limb dynamics
Source: PLoS One. 2025 Apr 30;20(4):e0322092. doi: 10.1371/journal.pone.0322092 (PMC12043163; doi:10.1371/journal.pone.0322092)
Supplement: Attachment — Submitted filename: Response to Reviewers.pdf [file pone.0322092.s009.pdf]

Reviewer #1:

In this paper authors explore the effect of biomechanical constraints on the dimensionality of control space. They showed that the muscle torques that support the limb against gravity are produced by more consistent combinations of muscle co-contraction than those that produce propulsion.

1. It is not clear to me why authors did PCA analysis of combined from different motor tasks data. If it were one motor task, it would show that CNS reduces control space to control this particular motor task. But what is possibly could be controlled for all different types of motions combined? Each motor task should be controlled differently. I would recommend to re-do analysis for each motor task separately.

Thank you for your comment. In this work, we have expanded upon our prior approach of analyzing reaching toward visual targets (Olesh et al. 2017 and Thomas et al. 2021). We used the PCA across movements in different directions to find the biomechanical underpinnings of muscle activation patterns which correspond to functions for supporting the limb against gravity and propulsion. Subdividing our dataset into separate movement directions and doing the PCA on each of them separately will result in the same 2 primary principal components but lower internal validity due to noise.

In response to the question of "what could be controlled for all different types of motions combined," the simple response is force or impedance are likely the parameters controlled by CNS. The idea of the modality of control signal has been discussed in the seminal works of Bizzi, Kalaska, Geogopoulus, Kawato, Wolpert, Shadmehr, Ting, and Gribble to name a few. These and other authors have developed influential theories of internal models, which accurately capture the sensorimotor transformations that occur in the CNS with the substantial predictive power of neural signals. We have expanded our description of these ideas in the Introduction on lines 38-48, 110-138, 148-163.

PCA can be thought of as a proxy for correlation analysis. Instead of examining each correlation separately, PCA does this across all correlations. The method includes computing the covariance matrix of the data first and then reducing it to a combination of scalars (eigenvalues) and vectors (eigenvectors), the latter pointing in the directions of the largest shared variances across the whole dataset. Thus, the scores or projections of the data onto the eigenvectors can tell us if the signals are changing together (positive correlations) or reciprocally (negative correlations). The sign of the scores is the indicator of that. That is why we did regressions between signed scores (not absolute values) to investigate the co-contraction across reaching in multiple directions. We have expanded our rationale for this in Methods on lines 279-301, 337-355, 375-393. We have added a new figure 4 to illustrate these points. We have also expanded on the signed

score amplitude analysis in Results, including statistical analysis, on lines 487 – 522 and show the score values in Figures 6,7,8 and Supplementary Figure S1 and S2.

Our assumption in this work is that principal components represent a low-dimensional control signal that can be produced as a result of neural activity. Some evidence supporting this assumption comes from neural recordings in non-human primates [59]. The known organization of neural circuits with multiple nested feedback loops provides clues to how the different co-contraction patterns can be produced by a reduced set of control signals. For example, the co-contraction of agonists, such as elbow flexors biceps, and brachioradialis, can be supported by common stretch reflex feedback from muscle spindles [60]. In this case, the co-contraction of agonists can be facilitated by a single descending signal that increases the gains of the stretch reflexes for these muscles. The negative reciprocal activation of antagonistic muscles, such as biceps and triceps, can be produced by inhibitory spinal interneurons, including Ia interneurons, that are known for their reciprocal inhibition action [61,62]. In this case, the reciprocal EMG can be facilitated by a single descending signal that excites the Ia interneurons for these muscles. The co-contraction of antagonistic action can be created through direct projections from the motor cortex, which is more extensive for motoneurons innervating distal muscles, through presynaptic inhibition of primary spindle afferents, the inhibition of Ia interneurons [63,64], or the spinal central pattern generator [37]. Although this analysis cannot distinguish between the contributions of different neural circuits to muscle recruitment, our method of analyzing the EMG co-contraction patterns can be used in future interventional studies, for example, with electrical or magnetic stimulation of specific neural structures, that can shed more light on the specific neural pathways that are involved in creating these patterns. We have added the descriptions of these possibilities on lines 619-639.

2. The authors wrote that there were 15 repetitions of each movement but in matrix A there is one EMG per motion. I suggest that analysis was done for each repetition separately, but it was not mentioned in the paper.

Although it is interesting to investigate the trial-to-trial variability, unfortunately, it is not possible to use our EMG data for this purpose. The EMG recordings were done using an older wired system with higher noise and motion artifacts. Only averaged EMG envelopes can be considered reliable as indicated by the EMG examples in Fig. 2C. The shaded areas show standard error, which is about 4 times smaller than the standard deviation for those traces. Furthermore, we are extending our previous conclusions from Olesh et al., 2017 to other conditions (different workspaces and to the non-dominant limb). Therefore, it is reasonable to apply the same analysis to the new data so that the results can be comparable.

3. The left part of Figure 1 is not necessary. Instead, it would be better to show locations of targets for different conditions, at least for one side.

Thank you for your suggestion, we have modified Figure 1 to show target locations for the right side.

4. Figure 2 has angles and angular velocities plotted on one panel. I would recommend using left/right different vertical axis as well using degrees instead of radians for angles.

Thank you for your suggestion, we have modified Figure 2 as requested.

5. pp.155-156 Authors wrote: “target was placed at a location that positioned the shoulder at 0 angle of all degrees of freedom and elbow at 90 degrees”. For some angles ‘0’ could be set in different positions. Was wrist pronated or supinated? Was wrist also parallel to the floor?

We have clarified the arm position description in Methods on lines 198-211. We have also included schematics of the arm position and targets in Fig. 1A to depict the DOFs at the central starting position for the lateral reaches.

6. pp.182-185 Authors wrote: “Joint angles representing 5 DOFs of the arm were derived using linear algebra, namely shoulder flexion-extension, shoulder abduction-adduction, shoulder internal-external rotation, elbow flexion-extension, forearm pronation-supination, and wrist flexion-extension”

   Are there 5 or 6 DOFs?
   shoulder flexion-extension(1), shoulder abduction-adduction(2), shoulder internal-external rotation(3), elbow flexion-extension(4), forearm pronation-supination(5), and wrist flexion-extension(6)

Thank you for identifying this error, we have corrected it. We incorrectly included forearm pronation-supination in the description. This degree of freedom was not included in the analysis in our earlier work. Thus, it was also not included in this analysis.

Reviewer #2:

Korol et al. examined muscle activity patterns during reaching movements in different directions and postures, performed by healthy individuals. They tested the relationship between muscle torques and muscle activity profiles, and the influence of biomechanical constraints on the dimensionality of control space. Using principal component analysis, they evaluated the contribution of individual muscles to joint torques. This work is an extension of a previous paper, the main novelty is that both dominant and non-dominant arm movements were included. They conclude that muscle torques supporting the limb against gravity are produced by more consistent

combinations of muscle co-contraction than those producing propulsion. There are several issues in the manuscript that need clarification. I list them in the following.

Major:

1. The paper is quite difficult to follow. This is mainly due to the writing style, use of many abbreviations, and sometimes confusing terminology (e.g. movement direction vs conditions of medial and lateral movements).

Thank you for identifying these issues. We have replaced "movement direction" with "reaching direction" to clarify that the motion from the central to one of the outward targets constituted reaching in an outward or inward direction. We are now consistently referring to 4 conditions as workspaces for reaching by either the left or right arm. We have reduced the number of abbreviations in the Results section by spelling out the terms.

2. EMG and torque was normalized to movement duration, yet movement durations were not reported. Heterogeneity in movement duration and speed of movement could significantly impact results.

Thank you for pointing this out. We have added the mean movement durations with standard deviations across participants in the Methods section (left lateral: 0.75 ± 0.10, left medial: 0.76 ± 0.06, right medial: 0.74 ± 0. 08, and right lateral: 0.71 ± 0.08 seconds). We have reported those on lines 261-263.

3. What is the rationale for including multiple repetitions and movement directions for each muscle in the matrix for the PCA? Is the assumption that center-out and return movements are driven by the same temporal synergies?

The data was averaged across multiple repetitions. The repetitions were done to make sure we obtained reliable profiles of kinematics and EMG. We used an older wired EMG system with more noise and motion artifacts than newer systems have. We have averaged across repetitions for each reaching direction, thus the PCA matrix included only the mean EMG profiles for each reaching direction (14 forward and 14 backward) for 12 muscles. Indeed, the assumption is that the outward and inward movements are examples of reaching movements in different directions from different starting locations and thus, would be driven by the same temporal synergies that are related to the underlying forces. We have clarified this on lines 264-265 and 337-355.

4. It is not clear why correlations of the scores of the first PCA component between muscles can be used as a proxy of co-activation or reciprocity. Other components likely contribute to the EMG and absolute values of the scores likely matter, i.e. two muscles with only positive scores can exhibit a negative correlation but that wouldn't mean that they are reciprocally activated. Why not simply calculate cross correlation of EMG directly?

PCA can be thought of as a proxy for correlation analysis. Instead of examining each correlation separately, PCA does this across all correlations. The method includes computing the covariance matrix of the data first and then reducing it to a combination of scalars (eigenvalues) and vectors (eigenvectors), the latter pointing in the directions of the largest shared variances across the whole dataset. Thus, the scores or projections of the data onto the eigenvectors can tell us if the signals are changing together (positive correlations) or reciprocally (negative correlations). The sign of the scores is the indicator of that. That is why we did regressions between signed scores (not absolute values) to investigate the co-contraction across reaching in multiple directions. We have expanded our rationale for this in Methods on lines 279-301, 337-355, 375-393. We have added a new figure 4 to illustrate these points. We have also expanded on the signed score amplitude analysis in Results, including statistical analysis, on lines 487 – 522 and show the score values in Figures 6,7,8 and Supplementary Figure S1 and S2.

The reviewer is correct in stating that the absolute values of the scores do matter as they are the magnitudes of the projection of each signal onto the eigenvector. We added a description of the implications of low PCA scores on lines 335-340 in Methods.

5. It is not clear if statistics were applied correctly. E.g. an RM-ANOVA has been applied to compare the coefficient of variation between conditions. It has not been stated if and how model assumptions were tested. It is very likely that the normality of residuals has been violated given the nature of the outcome variable. Furthermore, post-hoc tests were performed when the fixed effect was not significant. The presentation and description of the statistics is also lacking. It is at times not clear what model was calculated and test statistics should be reported.

The RM-ANOVA was applied to the coefficient of determination instead of the Pearson correlation coefficient because the former changes linearly and is normally distributed. We have added a description in Methods about verifying the ANOVA model assumptions in lines 315-323 We verified that the normality of residuals was met using Shapiro-Wilks test (custom function in MATLAB using *normcdf* and *norminv*). Due to multiple conditions included in ANOVA that can have different variances, sphericity was tested using Mauchly's test (part of *ranova* function). Sphericity was violated, so the Greenhouse-Geisser correction was used in the F and p statistics reported in Results. In addition, we have tested for outliers using the z-score method (*zscore* function in MATLAB). Using a threshold of 3 standard deviations, no outliers were detected.

Finally, we ensured equal sample sizes for R^2 across conditions.

We have expanded the description of the linear regression analysis, including correction for familywise error (alpha = 0.0008) on lines 375-393.

All F statistics, p values, and SE values from RM ANOVs are reported in the Results and Tables.

6. Equally, it is not clear if assumptions of the PCA were confirmed. E.g., are mean and covariance matrix sufficient to describe the distribution, are the intrinsic dimensions orthogonal, linearity of the low-dimensional manifold. Also, is normalization to the maximal EMG adequate? Would subtracting the mean followed by division by the standard deviation lead to different results? Peaks or baseline activity could skew the results.

We have added a description of how we addressed the PCA assumptions in Methods on lines 284-301.

We have normalized EMG to the maximal value for each muscle across reaching directions, which is a common practice in ensuring that the amplitude of the muscle activity scales within the 0 to 1 range. EMG's interval values of the original units of millivolts are not altered by this normalization. We also mean-centered our data before using PCA to avoid the PC1 capturing the mean of the data rather than the direction of maximum variance in the temporal profiles.

We have confirmed that the covariance matrix describes the distributions of EMGs and torques sufficiently well by using Shapiro-Wilk test for normality of residuals (alpha = 0.05). We have also confirmed that the intrinsic dimensions are orthogonal by computing the dot product of each pair of eigenvectors from EMG and torques. The off-diagonal elements of the dot product matrix were approximately zero (less than 10^{-10}), similar to an identity matrix, thus the principal components are orthogonal.

Due to the nature of EMG envelopes, it is expected that some muscles will have higher variance than others since certain reaching movements need more muscle activation than others. This meaningful heterogeneity reflects the inherent differences in the muscle activation patterns and forces they produce for different movement directions. The peaks of activity constitute meaningful variance that we want to include in our analysis. Division of the signals by the standard deviation will definitely skew the data, increasing the contribution of noise from the movements with low EMG and decreasing the contribution of meaningful variance from the movements with high EMG. The same logic applies to torques. While we acknowledge that ensuring homogeneous variance is an accepted technique in PCA, for reducing the dimensionality of EMG and torque signals in order to compare them afterward, we believe, it is not an appropriate data transformation.

7. Furthermore, it is intuitively not clear to me how temporal synergies with both positive and negative components and positive and negative scores could represent a neural signal driving multiple motor pools. How would the changes in signs be implemented on a circuit level? Wouldn't constraining scores to be non-negative result in synergies that are easier to interpret on a circuit level?

We have added a plot of all eigenvectors, showing that they have been aligned across all conditions and subjects (Figure 3). The procedure is now explained on lines 279-283. This ensured that there are no differences in sign between eigenvectors, the sign information is carried by the scores. We have expanded our rationale for relating positive and negative scores to co-contraction between muscles that is either positive, i.e. EMG signals increase or decrease together, or negative, i.e. some EMG signals increase, while others proportionally decrease, as in reciprocal activation of flexors and extensors. These clarifications are on lines 365-374 and 524-546.

Our assumption in this work is that principal components represent a low-dimensional control signal that can be produced as a result of neural activity. Some evidence supporting this assumption comes from neural recordings in non-human primates [59]. The known organization of neural circuits with multiple nested feedback loops provides clues to how the different co-contraction patterns can be produced by a reduced set of control signals. For example, the co-contraction of agonists, such as elbow flexors biceps, and brachioradialis, can be supported by common stretch reflex feedback from muscle spindles [60]. In this case, the co-contraction of agonists can be facilitated by a single descending signal that increases the gains of the stretch reflexes for these muscles. The negative reciprocal activation of antagonistic muscles, such as biceps and triceps, can be produced by inhibitory spinal interneurons, including Ia interneurons, that are known for their reciprocal inhibition action [61,62]. In this case, the reciprocal EMG can be facilitated by a single descending signal that excites the Ia interneurons for these muscles. The co-contraction of antagonistic action can be created through direct projections from the motor cortex, which is more extensive for motoneurons innervating distal muscles, through presynaptic inhibition of primary spindle afferents, the inhibition of Ia interneurons [63,64], or the spinal central pattern generator [37]. Although this analysis cannot distinguish between the contributions of different neural circuits to muscle recruitment, our method of analyzing the EMG co-contraction patterns can be used in future interventional studies, for example, with electrical or magnetic stimulation of specific neural structures, that can shed more light on the specific neural pathways that are involved in creating these patterns. We have added the descriptions of these possibilities on lines 619-639.

Minor:

1. Abstract: "Results generalize …" not clear that this refers to the previous work.

Thank you for your comment. We have rephrased the abstract to be more specific.

2. Line 62-65: There is quite some disagreement on whether PCA is adequate to identify synergies of EMG during locomotion. And this isn't the topic of the paper anyways. I'd remove this statement.

In these lines, we acknowledge that the PCA can be applied to the signals in different ways to obtain different types of synergies. The controversies are in whether these synergies capture the neural control signals that produce locomotion. Even without the concept of synergies, we know a lot about the neural circuitry controlling locomotion, such as central pattern generators and different types of spinal interneurons that couple motoneuron pools and transmit afferent feedback. That is why the example from locomotion showing that CPG can be captured by PCA when it is applied correctly makes a strong argument that PCA applied the same way to reaching EMG would give meaningful results. We have expanded the rationale for obtaining temporal synergies using PCA now on lines 65-93.

3. Lines 116-117: "We applied PCA with the assumption of invariant activation profiles to obtain temporal synergies" This needs more explanation.

We have expanded the description and rationale now on lines 110-138 and 144-163.

4. Lines 185-186: Calculation of active torques should be explained in this paper.

We have expanded our description of how the muscle torques were calculated now on lines 236-240 and 247-251.

5. LLat, RLat, etc. have not been defined.

These abbreviations have been removed throughout the text. They appear in some figure labels, defined in legend.

6. Lines 200-201: Rationale for filter settings is missing. (no low pass for the raw EMG, why 10 Hz for rectified).

We thank the reviewer for identifying this oversight. We have added a 500 Hz low-pass filter (line 257), re-filtered all raw EMGs, and re-ran all analyses. The overall results did not change, although the intra-subject variability reduced (lines 420-426) and the values of some PCA score correlations changed. All presented results are based on re-filtered EMG.

7. Line 244: not clear what hypothesis the authors refer to.

We have added earlier mentions of the hypothesis in the abstract and added a definition of it in the Introduction on lines 172-174.

8. Lines 300-301: We found that intra- and inter-subject variability of EMG profiles were very low" Average standard deviation of 12% of maximal EMG does not seem that low. Also not clear if this is intra or inter subject variability.

We have corrected this sentence and added values for both intra- and inter-subject variability on lines 420-426.

9. Lines 350-354: Fixed effect of sex was not reported; interaction effect neither. And for some reason comparison between sexes was reported as a post-hoc test with a p value of 0.06 and discussed as not significant.

We corrected this oversight and now report all main and interaction effects of the rmANOVAs. Statistics for correlations between eigenvectors is reported on lines 467-486 and in Tables 2 and 3. The effect of gender did become significant after EMG was re-filtered, we report it on lines 480-481. We have also added statistical analysis of PCA scores as now described in Methods on lines 337-355. The results of this analysis are reported on lines 487-522 and in Tables 4 and 5 and Supplementary Tables S1 and S2.

10. Lines 362-364: This conclusion is out of place and would need further elaboration.

We have removed this conclusion.
