# Supplementary material for: Control signal dimensionality depends on limb dynamics
Source: PLoS One. 2025 Apr 30;20(4):e0322092. doi: 10.1371/journal.pone.0322092 (PMC12043163; doi:10.1371/journal.pone.0322092)
Supplement: Attachment — Submitted filename: Reviewer reponses.docx [file pone.0322092.s010.docx]

Reviewer #1:

Question:

A very serious issue in the methods and results of the PCA analysis remains. I agree that “The idea of the modality of control signal has been discussed in the seminal works of Bizzi, Kalaska, Geogopoulus, Kawato, Wolpert, Shadmehr, Ting, and Gribble to name a few. These and other authors have developed influential theories of internal models, which accurately capture the sensorimotor transformations that occur in the CNS with the substantial predictive power of neural signals”.

Let me clarify my concern. CNS controls one movement at time, hence suggested modules also control one movement at a time. I also support the idea that common modules may be used to control the different movements. But this happens at different times. The authors can use PCA for particular movement to extract modules for this one movement, but if authors want to extract common modules involved in controlling different movements authors need to create matrixes Anxm by combining their each movement data sets into a matrix with number of muscles (12) as one dimension and larger time dimension (time(100) x number of movements (28) )(i.e., time dimension of the set up matrix is X28 larger, while first dimension is X28 smaller) and then using PCA to find common modules.

Surfacing the assumptions is another concern. The assumption is that there are the same two primary principal components of PCA analysis of separate movement directions for both EMG and torques. Authors didn’t show any evidence that this assumption is correct. And even if it is correct this is not the correct way to find common modules, as I mentioned above.

The correct estimation of a common modules is crucial for the results and interpretation and needs to be presented.

Answer:

We appreciate the reviewer’s clarifications. The PCA method described by the reviewer is aimed at obtaining spatial synergies, while our method is aimed at obtaining temporal synergies. We have had prior communications with Andrea d’Avella who described the differences between these two methods as follows: “In most muscle synergies studies, they [muscle synergies] are defined as coordinated activations of groups of muscles, i.e. as “spatial synergies”. This definition of muscle synergy is in line with that considered by the authors, i.e. as a control signals producing force in specific directions. Indeed, scaling the time-dependent and target-dependent activation of an invariant synergy one would expect that the generated force also scales. However, while not explicitly mentioned, in this paper PCA is used to extract “temporal synergies” rather than spatial synergies. **In this case, the synergies represent invariant temporal profiles that reconstruct the data through muscle-dependent and target-dependent scores**.” Both types of analyses have been utilized in prior EMG studies and provide different information about the neural control mechanisms (Brambilla et al. 2023). Our introduction expands on this point as follows: “The commonly used Principal Component Analysis (PCA) can be applied across muscles, experimental conditions, and time in two ways, to obtain temporally-variable synergies (temporal synergies) or temporally invariant synergies (spatial synergies) A recent study comparing spatial and temporal synergies has shown that these two types of synergies capture different features in EMG, which potentially reflect different aspects of neural control generating them [31]. Our earlier work shows that the biomechanical dynamics during reaching is captured accurately by the temporal synergies obtained with PCA. Specifically, the temporal dynamics of muscle torques, defined as rotational forces about joints, that act to support the arm against gravity and propel it toward the goal are well described by the 1^st^ and 2^nd^ temporal synergies, respectively, obtained with PCA [32]. Thus, the PCA method that obtains temporal synergies ensures that the forces needed to move the limb or limb dynamics are reflected in the signals with reduced dimensionality. Arguably, these signals are more likely to be represented at the neural circuit level because of the idea of the neural embedding of musculoskeletal dynamics, or internal models mentioned above, that enables the finite CNS circuitry to efficiently control movements in multiple directions from different starting postures at different speeds [33–35]. For example, the neural circuitry of the spinal central pattern generator that controls rhythmic behaviors, such as locomotion, by producing complex EMG patterns of multiple muscles spanning multiple joints is well understood [36–40]. A recent study has shown that the temporal dynamics of this spinal central pattern generator is captured well using temporal synergies obtained with PCA of EMG during locomotion [41]. This further proves that the temporal synergies obtained with PCA can accurately capture the known neural circuitry with complex temporal dynamics.”

Here we focus on temporal synergies because we have already established the relationship between these types of synergies and muscle torques in an earlier study (Olesh et al. 2016). This answers the second part of the question above about the assumptions. Our new study aims to extend the conclusions from the earlier study that established the relationship between EMG and muscle torques using temporal synergies obtained with PCA the same way to both limbs and to other workspace locations. In the Introduction we describe how our hypothesis is based on the prior studies as flows: “The temporal profile of the envelope of surface EMG is closely related to the force the corresponding muscle is producing in response to neural activation [42]. More recently we have shown that for reaches with the dominant hand towards visual targets in three-dimensional space, the EMG profiles are closely related to the muscle torques that cause motion [32]. Specifically, the static component of EMG that underlies postural forces needed to support the arm in a specific posture and during transitions between postures is closely related to the components of muscle torques that include gravity terms in the equations of motion. This static component of EMG can be extracted as the 1^st^ principal component of PCA applied to obtain temporal synergies [32]. This indicates that the static component of the EMG envelope from a given muscle reflects the muscle’s contribution to counteracting gravity load on the joints it spans. Moreover, the residual phasic component of EMG is closely related to the residual muscle torque that underlies the acceleration and deceleration forces toward the reaching goal after the gravity-related component is subtracted. This phasic component of EMG can be extracted as the 2^nd^ principal component of PCA [32]. This indicates that the phasic component of the EMG envelope from a given muscle reflects the muscle’s contribution to propulsion toward the target and stopping there. Here we will test the generalizability of these relationships between muscle activity and torque profiles to the non-dominant limb and across different workspaces.”
